# Intelligent Fault Diagnosis of Rolling Bearings Based on a Complete Frequency Range Feature Extraction and Combined Feature Selection Methodology

**DOI:** 10.3390/s23218767

**Published:** 2023-10-27

**Authors:** Zhengkun Xue, Yukun Huang, Wanyang Zhang, Jinchuan Shi, Huageng Luo

**Affiliations:** School of Aerospace Engineering, Xiamen University, Xiamen 361102, China; xuezhengkun@stu.xmu.edu.cn (Z.X.); huangyukun@stu.xmu.edu.cn (Y.H.); 34720230156528@stu.xmu.edu.cn (W.Z.); shijinchuan@stu.xmu.edu.cn (J.S.)

**Keywords:** rolling bearing fault diagnosis, fuzzy dispersion entropy, feature extraction, feature selection

## Abstract

The utilization of multiscale entropy methods to characterize vibration signals has proven to be promising in intelligent diagnosis of mechanical equipment. However, in the current multiscale entropy methods, only the information in the low-frequency range is utilized and the information in the high-frequency range is discarded. In order to take full advantage of the information, in this paper, a fault feature extraction method utilizing the bidirectional composite coarse-graining process with fuzzy dispersion entropy is proposed. To avoid the redundancy of the full frequency range feature information, the Random Forest algorithm combined with the Maximum Relevance Minimum Redundancy algorithm is applied to feature selection. Together with the K-nearest neighbor classifier, a rolling bearing intelligent diagnosis framework is constructed. The effectiveness of the proposed framework is evaluated by a numerical simulation and two experimental examples. The validation results demonstrate that the extracted features by the proposed method are highly sensitive to the bearing health conditions compared with hierarchical fuzzy dispersion entropy, composite multiscale fuzzy dispersion entropy, multiscale fuzzy dispersion entropy, multiscale dispersion entropy, multiscale permutation entropy, and multiscale sample entropy. In addition, the proposed method is able to identify the fault categories and health states of rolling bearings simultaneously. The proposed damage detection methodology provides a new and better framework for intelligent fault diagnosis of rolling bearings in rotating machinery.

## 1. Introduction

In modern industrial systems involved in major engineering fields such as aviation, electric power, the chemical industry, and mining, rotating machinery has been widely used as an integral part of such systems. With the continuous progress of modern science and technology, the complexity of rotating machinery systems is also getting higher [1]. The rolling bearing, as a key component, usually operates continuously in a harsh environment and under complex loading; thus, it is prone to failures [2,3]. Once a rolling bearing failure occurs, it will directly affect the reliability and the stability of the rotating machinery system, and it sometimes produces safety risks or even leads to catastrophic accidents [4]. Therefore, the research of rolling bearing health condition monitoring and intelligent fault diagnosis is very important.

Due to its cost-effectiveness, the vibration sensor has been widely used in bearing condition monitoring [5]. In general, vibration-based rolling bearing health condition monitoring and intelligent fault diagnosis technology consists of three major steps: vibration signal data acquisition, signal feature extraction, and fault identification and classification [6,7]. Among these steps, the process of feature extraction will greatly affect the final identification. The extraction of proper features from the complex vibration signals is the key step to realizing intelligent fault diagnosis [8,9].

As a statistical measure, information entropy can be used to quantify the internal information and complexity of a time series [10]. Usually, the greater the complexity or irregularity of the time series, the larger the corresponding entropy value. In contrast, a smaller entropy value usually means less complexity and less irregularity in the time series. As is well known, the introduction of faults in a bearing will increase the complexity of the vibration signal during operations; therefore, an appropriately configurated entropy could be a feasible measure for the bearing health conditions.

Based on vibration signals, some commonly defined information entropy has been successfully applied to the field of fault diagnosis and has yielded useful results. For example, Yan [11] used the approximate entropy as the feature index of signal complexity measurement and successfully applied it to the identification of structural defects in bearings. Han et al. [12] adopted the sample entropy as an index to reflect the regularity and features of vibration signals and realized the fault diagnosis of rolling bearings. Zheng et al. [13] utilized the fuzzy entropy as the feature and demonstrated its effectiveness through experimental verifications. Zhang et al. [14] characterized the fault state of motor bearings by using the permutation entropy of the vibration signal. The effectiveness of their method was verified through experiments and comparative studies. Rostaghi et al. [15] proposed dispersion entropy for condition monitoring of rotating machinery and, through several sets of experimental data, demonstrated that it has a better effect than other usual entropy methods. In recent years, there have also been efforts made towards improvements of the entropy methods based on dispersive entropy which have been applied to the field of fault diagnosis [16,17].

For more complicated diagnosis situations, the above-mentioned single-scale entropy is sometimes not sufficient. Accordingly, the multiscale entropy method has been proposed [18]. Multiscale entropy is calculated through expanding the original time series into a multiple scale series by using the coarse-graining method. Long et al. [19] realized rolling bearing different fault category diagnosis by using the multiscale sample entropy calculated from the vibration signal as the feature index and compared it with the results of using a traditional single-scale sample entropy as the index. Wu et al. [20] extracted the features of multiscale permutation entropy from the vibration signals of faulty rolling bearings and compared them with that of single-scale permutation entropy and multiscale sample entropy methods to verify the superiority and effectiveness of the multiscale permutation entropy method. Zhang et al. [21] utilized the multiscale dispersion entropy of the vibration signal as the feature for rolling bearing diagnosis and obtained an effective diagnosis result.

Multiscale-based entropy feature extraction methods are widely used, but they still have two major drawbacks. From one aspect, multiscale entropy based on the traditional coarse-graining method has a large variance of entropy value when the scale is large, causing the reliability of the entropy assessment results to be reduced. On the other side, the current multiscale methods extract only the low-frequency information from the sequence, without considering the high-frequency information. In order to overcome the problem of a high variance of entropy value in multiscale entropy methods based on traditional coarse-graining methods, composite multiscale entropy was proposed [22]. Although the problem of a high variance of entropy value at multiscale was solved, the issue of utilizing the high-frequency information has not yet been resolved. Hierarchical entropy [23] is considered as an entropy method that takes into account the high-frequency information of a sequence and essentially coarse-grains the sequence using two operators—difference and average. However, hierarchical entropy is unable to consider multiscale information concurrently, and the coarse-grained sequences in different layers do not distribute as high-frequency components or low-frequency components [24]. In addition, after the multiscale entropy feature extraction of the sequences, the feature selection was not considered in most cases, which could create the problem of redundancy of feature information. Therefore, it has become necessary to introduce a new method which is able to take advantage of the full frequency information and minimize possible feature redundancy.

The fuzzy dispersion entropy (FDE) has been proven to be a promising method for feature extraction [25]. However, it also suffers from the drawback of a single scale, which makes it difficult to reflect information from multiple scales. To fully extract the entropy feature information of the full frequency band of the signal while avoiding feature information redundancy and achieving better diagnosis, an intelligent diagnosis framework for rolling bearing fault identification based on a combination of the bidirectional composite multiscale fuzzy dispersion entropy (BCMFDE), Random Forest (RF) algorithm, Maximum Relevance Minimum Redundancy (mRMR) algorithm, and the K-nearest neighbor (KNN) classifier is proposed. A numerical simulation and two experiments are used to demonstrate the effectiveness and versatility of the proposed method.

The rest of this paper is organized as follows. Section 2 describes the basic definitions of the proposed methodology. Section 3 describes the framework of the proposed fault diagnosis method. Section 4 simulates the signals of different faults of rolling bearings and verifies the effectiveness of the proposed method using the simulated signals. Section 5 verifies the effectiveness of the proposed method by two experimental examples. Section 6 summarizes the conclusions.

## 2. Methods

### 2.1. BCMFDE

The BCMFDE method is formed by combining the FDE method and the bidirectional composite coarse-graining process (BCCGP).

#### 2.1.1. FDE

The FDE is a method used to characterize the complexity of a time series and estimate the dynamic changes of signal fluctuations. The calculation procedure is as follows [26]:

1. The time series x={x(i), i=1,2,…,N} is mapped to y={y(i), i=1,2,…,N} through the Normal Cumulative Distribution Function (NCDF). Each element in vector y is defined as:

(1) $$y\left(i\right)=\frac{1}{\sigma \sqrt{2\pi }}\int_{-\infty }^{x(i)}e^{\frac{{-(t-\mu )}^{2}}{2\sigma^{2}}}dt$$

where i=1,2,…,N, y(i)∈(0,1), σ and μ are the standard deviation and the expectation of x, respectively, and N is the number of data points.

2. Each element y(i) is mapped to a new symbolic sequence $Z^{c}=\{z_{1}^{c},z_{2}^{c},\ldots ,z_{N}^{c}\}$ using a linear transformation as follows:

(2) $$z_{i}^{c}=c\cdot y(i)+0.5$$

where c is the number of categories.

3. The series $Z^{c}$ with the embedding dimension m and the delay time d are constructed as follows:

(3) $$z_{j}^{m,c}=\{z_{j}^{c},z_{j+(1)d}^{c},\cdots ,z_{j+(m-1)d}^{c}\},j=1,2,\cdots ,N-(m+1)d$$

4. The fuzzy membership function is introduced in sequence $Z^{c}$ as follows:

(4) $$\mu M_{1}(z_{i}^{c})=\left\{\begin{array}{c} 0\\ 2-z_{i}^{c}\\ 1 \end{array}\right.\begin{array}{c} z_{i}^{c}>2\\ 1\leq z_{i}^{c}\leq 2\\ z_{i}^{c}<1 \end{array}$$

(5) $$\mu M_{k}(z_{i}^{c})=\left\{\begin{array}{c} \begin{array}{ccc} 0 & & z_{i}^{c}>k+1 \end{array}\\ \begin{array}{c} \begin{array}{ccc} k+1-z_{i}^{c} & & k\leq z_{i}^{c}\leq k+1 \end{array}\\ \begin{array}{c} \begin{array}{ccc} z_{i}^{c}-k+1 & & k-1\leq z_{i}^{c}\leq k \end{array}\\ \begin{array}{ccc} 0 & & z_{i}^{c}<k \end{array}-1 \end{array} \end{array} \end{array}\right.$$

(6) $$\mu M_{c}(z_{i}^{c})=\left\{\begin{array}{c} 1\\ z_{i}^{c}-c+1\\ 0 \end{array}\right.\begin{array}{c} z_{i}^{c}>c\\ c-1\leq z_{i}^{c}\leq c\\ z_{i}^{c}<c-1 \end{array}$$

5. Each vector $z_{j}^{m,c}$ is mapped to a dispersion pattern $\pi_{v_{0},v_{1},\ldots ,v_{\left(m-1\right)}}$ according to its degrees of membership. where $z_{j}^{c}$ is class $v_{0}$, $z_{j+(1)d}^{c}$ is class $v_{1}$,…, and $z_{j+(m-1)d}^{c}$ is class $v_{m-1}$. The membership degree of each vector $z_{j}^{m,c}$ is calculated to obtain the membership degree of each dispersion pattern:

(7) $$\mu_{\pi_{v_{0},v_{1},\ldots ,v_{m-1}}}\left(z_{j}^{m,c}\right)=\prod_{i=0}^{m-1}\mu M_{vi}(z_{j+(i)d}^{c})$$

In general, the number of dispersion patterns that are attributed to each vector $z_{j}^{m,c}$ in FDE is equal to $c^{m}$.

6. The probability of each dispersion pattern $\pi_{v_{0},v_{1},\ldots ,v_{m-1}}$ is calculated as follows:

(8) $$p(\pi_{v_{0},v_{1},\ldots ,v_{m-1}})=\frac{\sum_{j=1}^{N-(m-1)d}\mu_{\pi_{v_{0},v_{1},\ldots ,v_{m-1}}}(z_{j}^{m,c})}{N-(m-1)d}$$

7. Finally, the FDE is calculated according to the theory of Shannon’s entropy as follows:

(9) $$FDE(x,\;m,\;c,\;d)=-\sum_{\pi =1}^{c^{m}}p(\pi_{v_{0},v_{1},\ldots ,v_{m-1}})ln(p(\pi_{v_{0},v_{1},\ldots ,v_{m-1}}))$$

#### 2.1.2. BCCGP

The BCCGP is based on an improvement of the composite coarse-graining process [27], which has the advantage of making BCCGP capable of dealing with the multiscale decomposition of the low- and high-frequency components of the time series. The calculation procedure of the BCCGP is as follows:

1. For time series x={x(i); i=1,2,…,N} of length, N is a positive integer, and the bidirectional composite coarse-graining operator at τ scales factors is expressed as

(10) $$\left\{\begin{array}{c} d_{o,j}^{\left(\tau \right)}=x(\tau \times j+(o-1))-\frac{1}{\tau -1}\sum_{f=0}^{\tau -2}x(\tau (j-1)+o+f),j=1,2,\ldots ,\left\lfloor \frac{N}{\tau }\right\rfloor ,o=1,2,\ldots ,\tau .\\ a_{o,j}^{\left(\tau \right)}=\frac{1}{\tau }\sum_{f}^{\tau }x(\tau (j-1)+o+f),j=1,2,\ldots ,\left\lfloor \frac{N}{\tau }\right\rfloor ,o=1,2,\ldots ,\tau . \end{array}\right.$$

where $d_{o,j}^{\left(\tau \right)}$ and $a_{o,j}^{\left(\tau \right)}$ represent for difference operators and average operators, respectively.

2. The coarse-grained series form of operators $d_{o,j}^{\left(\tau \right)}$ and $a_{o,j}^{\left(\tau \right)}$ is expressed as:

(11) $$\left\{\begin{array}{c} H_{o}^{\left(\tau \right)}=\left\{d_{o,1}^{\left(\tau \right)},d_{o,2}^{\left(\tau \right)},\ldots ,d_{o,j}^{\left(\tau \right)}\right\}\\ L_{o}^{\left(\tau \right)}=\left\{a_{o,1}^{\left(\tau \right)},a_{o,2}^{\left(\tau \right)},\ldots ,a_{o,j}^{\left(\tau \right)}\right\} \end{array}\right.$$

where $H_{o}^{\left(\tau \right)}$ and $L_{o}^{\left(\tau \right)}$ represent the coarse-grained series for high-frequency components and low-frequency components, respectively.

3. According to the definition of FDE, the BCMFDE is obtained by

(12) $$BCMFDE(x,m,c,d,\tau )=\frac{1}{\tau }\sum_{o}^{\tau }FDE(d_{o,j}^{\left(\tau \right)},m,c,d),\frac{1}{\tau }\sum_{o}^{\tau }FDE(a_{o,j}^{\left(\tau \right)},m,c,d)$$

**Figure 1:**
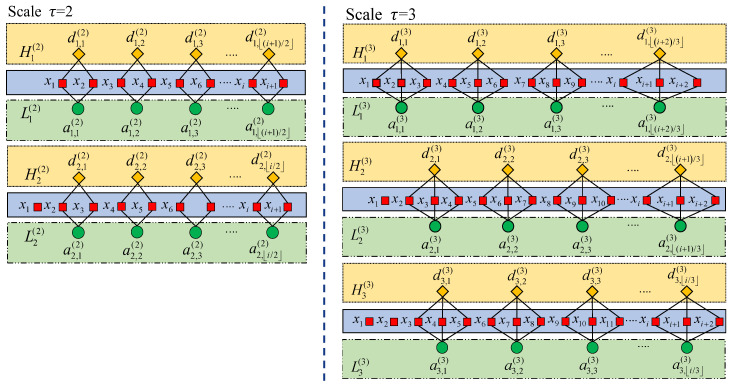
Examples of BCCGP.

The BCCGP with scale factors τ=2 and τ=3 is shown in Figure 1. In the process of bidirectional composite coarse-graining, the difference and average operators are used to process the original time series, so the BCCGP is capable of capturing more comprehensive time series feature information from the low-frequency components and the high-frequency components of the time series simultaneously.

### 2.2. Feature Selection

The advantage of BCMFDE is that the information of low- and high-frequency components at different scales is considered. However, the more sequence scales that are considered, the more information features that can be constructed, and consequently, the more computational effort that is required. To balance feature richness and the computational burden, it is necessary to make a reasonable selection of the extracted feature set. To this end, the RF-mRMR is used for feature selection.

#### 2.2.1. RF

The RF is an ensemble learning algorithm composed of decision tree models as its basic units [28]. The essence of the algorithm is generated by integrating the results from all decision tree models and determining the final result with votes. The RF algorithm is capable of evaluating the importance of features, and the main idea is to calculate the contribution of different features to each decision tree model. The contribution is able to be represented using the calculation of the out-of-bag (OOB) data error rate [29], where the OOB data are unused data each time the decision tree is built. The importance of the features is measured by calculating the average contribution of each feature.

To evaluate the importance of a feature, the steps are as follows:

1. The corresponding OOB data are selected for each decision tree to calculate the OOB data error rate, denoted as eOOB1.
2. The OOB data error rate is calculated again after adding random noise interference to all samples of OOB data and is denoted as eOOB2.
3. The importance ψ of the feature when there are $N_{t}$ decision trees in the forest can be expressed as:

(13) $$\psi =\frac{\sum_{i=1}^{N_{t}}eOOB1\left(i\right)-eOOB2\left(i\right)}{N_{t}}$$

The reason that ψ reflects the importance of the feature is that when random noise is added, the accuracy of the OOB data decreases sharply (eOOB2 increases), which indicates that the feature has a major impact on the model results and that the importance of the feature is relatively significant. The parameters of RF are set based on the suggestions given in [30], where the decision tree $N_{t}=50$.

#### 2.2.2. mRMR

The mRMR is acting as a filter for feature evaluation and selection [31,32]. The core concept of the mRMR algorithm is to maximize the relevance between features and categorical variables while minimizing the redundancy between different features. The basic theory of the mRMR algorithm is summarized below [33].

The mutual information amount is used to measure the similarity between variables, and the corresponding score is assigned to achieve feature selection according to the size of the score. For the two given random variables X and Y, their mutual information is defined as:

(14) $$I(X;Y)=\sum_{a\in X}\sum_{b\in Y}p(a,b)log\frac{p(a,b)}{p(a)p(b)}$$

where p(a) and p(b) represent the probabilities of X and Y, respectively, and p(a,b) is the joint probability density function of X and Y.

Assume that $T_{i}$ represents each feature and that v represents a category. In order to ensure the maximum relevance between the feature and the category, the maximum relevance criterion is expressed as:

(15) $$\max D(S,v),D=\frac{1}{\left|S\right|}\sum_{T_{i}\in S}I(T_{i};v)$$

where S is a feature subset, and $I(T_{i};v)$ is the mutual information between features $T_{i}$ in different categories v.

The defined maximum relevance criterion is able to find the feature subset S that has the greatest information correlation with each type of feature; however, the feature subset selected based on this criterion may have redundancy. In order to ensure minimum redundancy among features, the minimum redundancy criterion needs to be applied. The minimum redundancy criterion is expressed as:

(16) $$\min R(S),R=\frac{1}{{\left|S\right|}^{2}}\sum_{T_{i},T_{j}\in S}I(T_{i};T_{j})$$

In order to retrieve the feature sets with maximum relevance and minimum redundancy, D and R need to be optimized simultaneously. To achieve this, we define a feature sensitivity Φ for each feature as:

(17) $$\max\Phi (D,R),\Phi =\frac{D}{R}$$

### 2.3. KNN

The KNN [34] classifier, which is widely used in mechanical fault diagnosis, was used to achieve the classification of rolling bearings with different types and severity of faults.

The KNN classifier is used for classification by measuring the distance between different feature data. The basic idea is that a sample h is assumed to have K nearest neighboring samples $h_{K}$ in the feature space. If most of the samples $h_{K}$ belong to category L, then, that sample h also belongs to category L.

The basic steps of KNN include:

1. Calculating the distance between the feature data of the test sample and the feature data of each training sample.
2. Ranking the distance according to its magnitude.
3. Selecting the K samples with the smallest distance.
4. Calculating the frequency of occurrence of the category in which the top K samples are located.
5. Returning the category with the highest occurrence frequency among the top K samples as the classification of the test sample.

**Figure 2:**
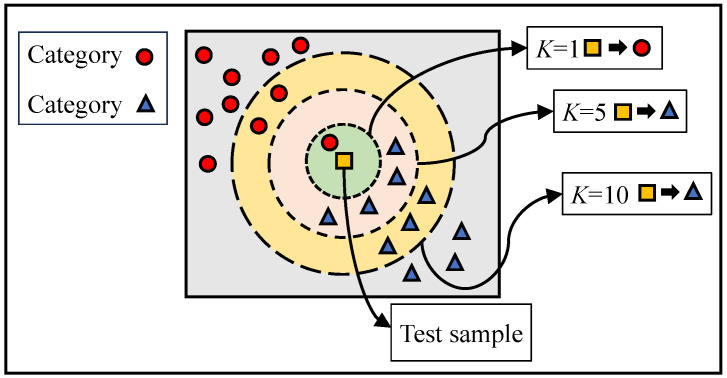
Schematic diagram of KNN classifier.

The value of the nearest neighbor number *K* affects the results of the model, as shown in Figure 2. As shown in Figure 2, the judgment results under K=5 or K=10 are inconsistent with those under K=1. This indicates that the number of nearest neighbors K affects the complexity and generalization of the model. Therefore, in order to make the model have better generalization, in this paper, K=5 is chosen as the number of nearest neighbors for the KNN classifier.

## 3. Intelligent Fault Diagnosis Framework

**Figure 3:**
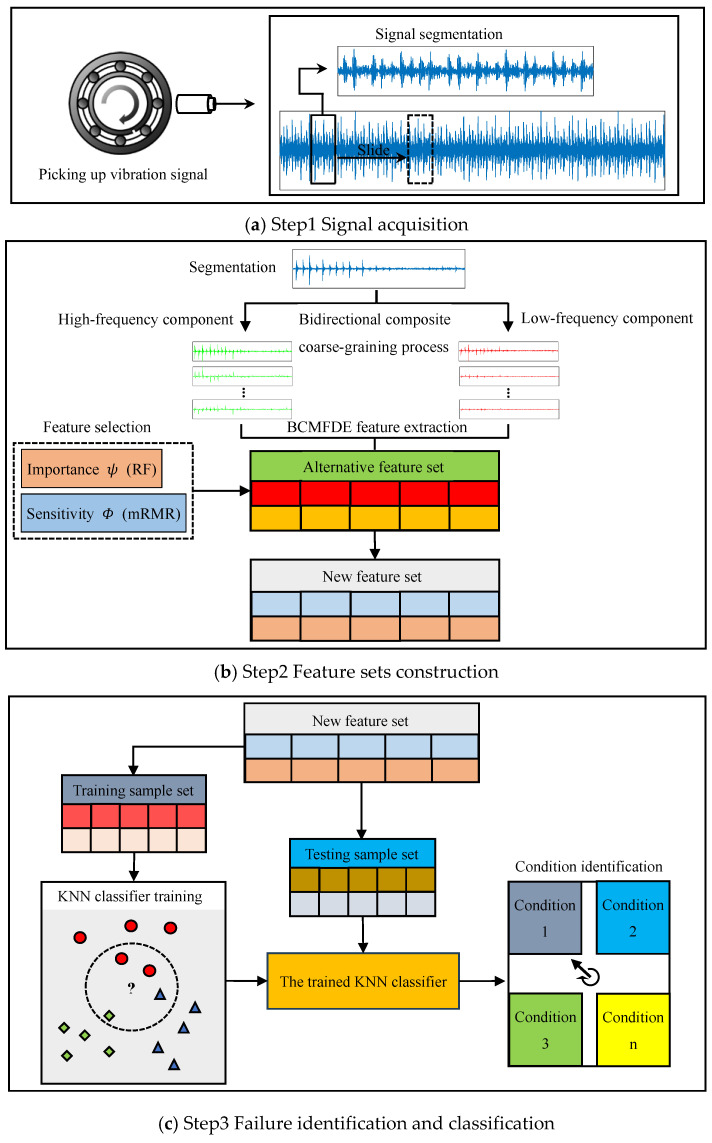
Schematic diagram of the intelligent fault diagnosis framework.

Based on the above discussions, we propose a rolling bearing fault feature sets construction method based on the BCMFDE feature extraction method combined with the RF-mRMR feature selection method, aiming at extracting features in the whole frequency range of the signal while minimizing the information redundancy. And, it is combined with the KNN classifier to form an intelligent fault diagnosis framework. The procedures of the proposed intelligent fault diagnosis framework are depicted in Figure 3, which include the following steps:

- Step 1: Signal acquisition as shown in Figure 3a. The vibration sensor is used to collect the dynamic response for bearing condition diagnosis. The collected vibration signal is segmented with equal length before the signal being analyzed.
- Step 2: Feature sets construction as shown in Figure 3b. Firstly, the analyzed signal is subjected to BCCGP processing to obtain the low-frequency and high-frequency component series in different scales. The FDE of each series is calculated using Equation (12). The alternative feature set of rolling bearing faults consisting of BCMFDE is constructed. Secondly, the RF-mRMR is used to select the dominant features from the rolling bearing alternative feature set based on the importance ψ and sensitivity Φ of features at each scale to obtain a new rolling bearing fault feature set.
- Step 3: Failure identification and classification as shown in Figure 3c. The new rolling bearing fault feature set is randomly divided into a training sample set and a test sample set. The training sample set is used to train the KNN classifier. The test samples are used as input to the trained KNN classifier to test the classifier’s ability to identify rolling bearing health conditions in rotating machinery.

## 4. Simulation

### 4.1. Simulated Bearing Damage Vibration Response

**Figure 4:**
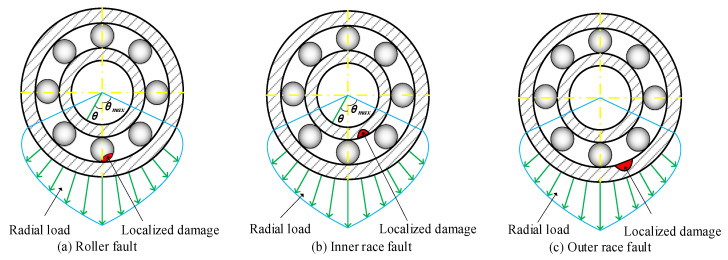
Schematic diagram simulating rolling bearing state.

In this section, simulated signals of rolling bearings with different faults are used to evaluate the effectiveness of the proposed signal processing framework [35]. Simulated bearing faults include: the roller fault, the inner race fault, and the outer race fault. The sampling frequency is 10.24 kHz. The shaft rotating speed is 1800 rpm. The bearing parameters are listed in Table 1. The schematic diagram of the simulated bearing is shown in Figure 4.

Assuming at time t=0, the local defect begins to make contact with a roller. The impact force excited by the local defect on the outer ring can be expressed as:

(18) $$D_{o}(t)=\sum_{t=-\infty }^{+\infty }d_{o}\delta (t-\frac{k}{f_{o}})$$

Then, the impact force excited by the local defect on the inner ring can be expressed as:

(19) $$D_{i}(t)=\sum_{t=-\infty }^{+\infty }d_{i}\delta (t-\frac{k}{f_{i}})$$

and the impact force excited by the local defect on the roller can be expressed as:

(20) $$D_{b}(t)=\sum_{t=-\infty }^{+\infty }d_{bo}\delta (t-\frac{k}{f_{b}})+\sum_{t=-\infty }^{+\infty }d_{bi}\delta (t-\frac{k-\beta }{f_{b}})$$

In Equations (18)–(20), $d_{o}$ represents the pulse intensity of the outer race; $d_{i}$ is the inner race pulse intensity; $d_{bo}$ is the pulse intensity of the outer race to the roller; $d_{bi}$ is the pulse intensity of the inner race to the roller; δ is the unit impulse function; k is the number of pulses; β is the pulse phase difference coefficient, which is β=0.5; $f_{o}$ is the characteristic frequency of the bearing outer race damage; $f_{i}$ is the characteristic frequency of the bearing inner race damage; and $f_{b}$ is the characteristic frequency of the roller damage. For simplicity, the pulse intensity in the simulation is assumed to be unity.

The vibration amplitude decay envelope function due to damping can be expressed as:

(21) $$e(t)=\left\{\begin{array}{c} e^{-2\pi \zeta_{e}f_{e}t}\\ 0 \end{array}\;\;\;\;\right.\begin{array}{c} t>0\\ t\leq 0 \end{array}$$

where $\zeta_{e}$ and $f_{e}$ are the damping ratio and the natural frequency of the bearing system, respectively.

The amplitude transfer function is expressed as:

(22) p(θ)=cos(θ)

The load distribution can be expressed as:

(23) $$q(\theta )={q_{max}[1-(1/2\varepsilon )(1-cos\theta )]}^{n}$$

where $q_{max}$ represents the maximum load intensity, and ε represents the load distribution coefficient.

The simulated outer race fault signal can be expressed as:

(24) $$x_{o}(t)=D_{o}(t)*e(t)$$

The simulated inner race fault signal can be expressed as:

(25) $$x_{i}(t)=D_{i}(t)q(2\pi f_{r}t)p(2\pi f_{r}t)*e(t)$$

where $f_{r}$ is the rotating frequency of the shaft.

The simulated roller fault signal can be expressed as:

(26) $$x_{b}(t)=D_{b}(t)q(2\pi f_{c}t)p(2\pi f_{c}t)*e(t)$$

where $f_{c}$ is the rotating frequency of the bearing cage.

**Figure 5:**
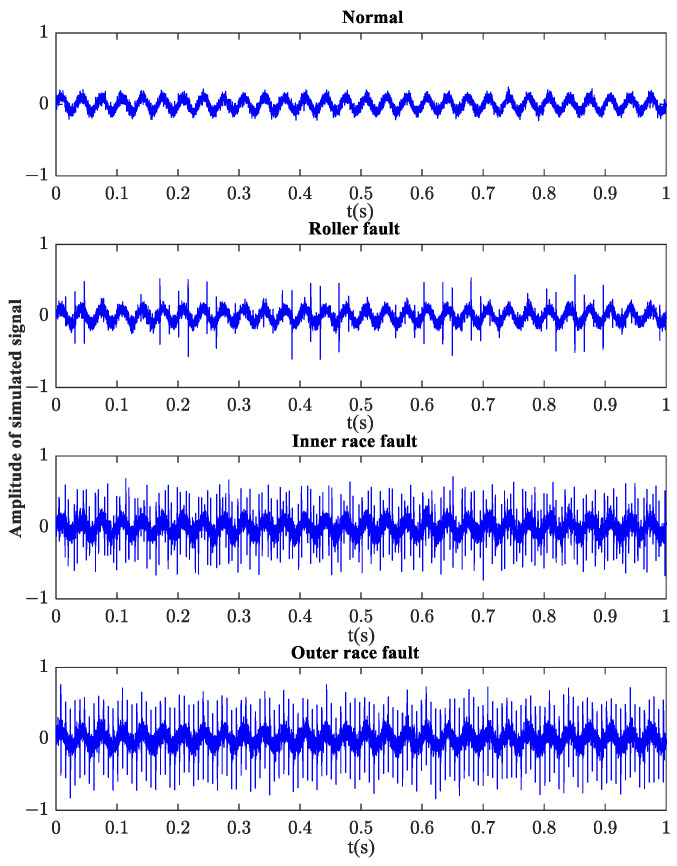
Simulated vibration signals with different bearing states.

In order to simulate the actual working conditions, Gaussian white noise is added into the analog signal, and the signal noise ratio is 5 dB. The time history of the simulated bearing vibration response with normal, roller fault, inner race fault, and outer race fault are plotted in Figure 5.

### 4.2. Simulation Analysis

The vibration signal is processed according to the procedures outlined in Section 3. Based on suggestions given in [27], for each vibration response, a sliding window of 2048 points is applied to extract 300 samples from the original signal. The BCMFDE features are extracted for each sample to construct the feature set. For the purpose of comparisons, other entropy feature extraction methods are also applied to the same signal, including HFDE (Hierarchical fuzzy dispersion entropy), CMFDE (Composite multiscale fuzzy dispersion entropy), MFDE (Multiscale fuzzy dispersion entropy), MDE (Multiscale dispersion entropy), MPE (Multiscale permutation entropy) and MSE (Multiscale sample entropy). Based on suggestions given in [25,36], the parameters used for feature calculation are listed in Table 2.

**Figure 6:**
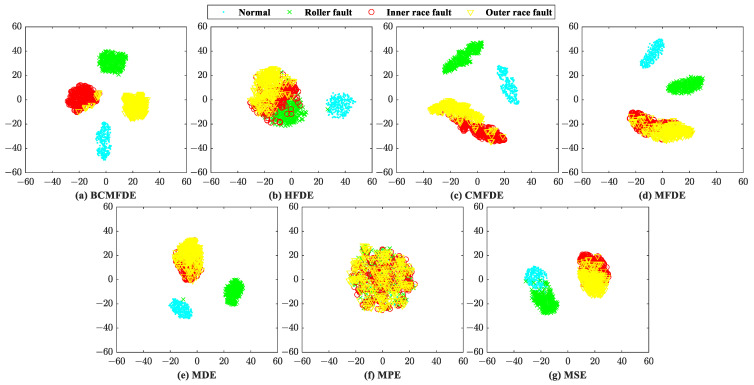
Feature set visualization for different entropy methods.

The feature sets constructed by different entropy methods are visualized by using the t-SNE [37] algorithm first, as shown in Figure 6. According to [30], in a feature set, when the distance between samples of the same category is small and the distance between samples of different categories is large, it indicates that the constructed feature set has good separability for feature categorization. Therefore, judging by visualization, from Figure 6, the feature set constructed by the BCMFDE method has the best separability among the methods used.

In order to quantitatively evaluate the feature extraction ability of different entropy methods, the classification effect of the feature set was tested. The KNN classifier was used in the evaluation, and the test accuracy was adopted as the evaluation measure.

The KNN classifier was trained first. Eighty percent of the feature sets were randomly selected to form the training set and the rest were used as the test set. The training sets were used to train the classifier model first. The test sets were then fed to the trained model to validate the classification accuracy. This process was repeated 10 times and the mean, standard deviation, and mean time values of the test accuracies were calculated and listed in Table 3 for comparisons.

It can be seen from Table 3 that among the MFDE, MDE, MPE, and MSE methods, the MFDE method obtained the highest mean accuracy and the smallest standard deviation. This indicates that the sensitivity of FDE features is higher. In addition, feature extraction based on BCMFDE produced the best mean accuracy and standard deviation compared with the other traditional coarse-grained methods, indicating that the bidirectional composite coarse-graining-based approach indeed increased information richness and, therefore, provided better classification accuracy.

**Figure 7:**
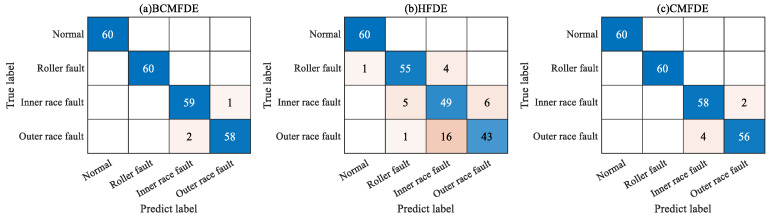

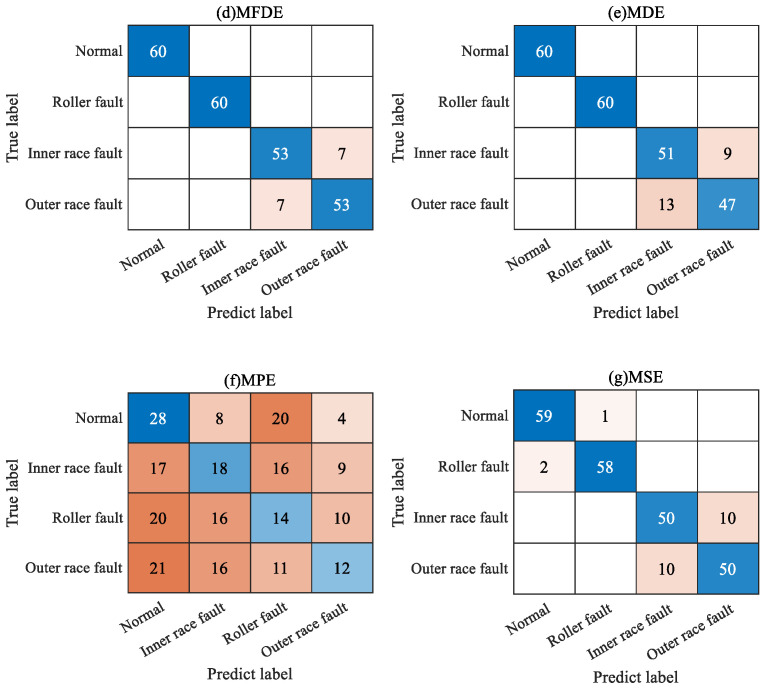
Confusion matrix of different entropy methods.

The confusion matrix can intuitively show the category and number of samples that were misclassified. The confusion matrix of the fifth test result was visualized and analyzed, as shown in Figure 7. It can be seen from Figure 7 that in the feature set constructed using the BCMFDE method, the number of misclassified samples is the smallest. At the same time, it coincides with the t-SNE visualization results of the feature set in Figure 6. This indicates that the BCMFDE method has the best feature extraction capability.

Although the method based on BCMFDE improves classification accuracy, its disadvantages are an increased computational burden and the risk of feature information redundancy. Compared with traditional coarse-graining, bidirectional composite coarse-graining considers not only the information of low-frequency components but also the additional information of high-frequency components, which will double the number of extracted features. To avoid redundancy of feature information while minimizing the computation cost, the RF-mRMR is used to select important and sensitive features (assessed by importance ψ and sensitivity Φ and ranked from highest to lowest) from the raw feature set. The features with importance ψ and sensitivity Φ in the top τ are selected and then used to construct a new feature set. This feature selection procedure formulates the RF-mRMR–BCMFDE process.

**Figure 8:**
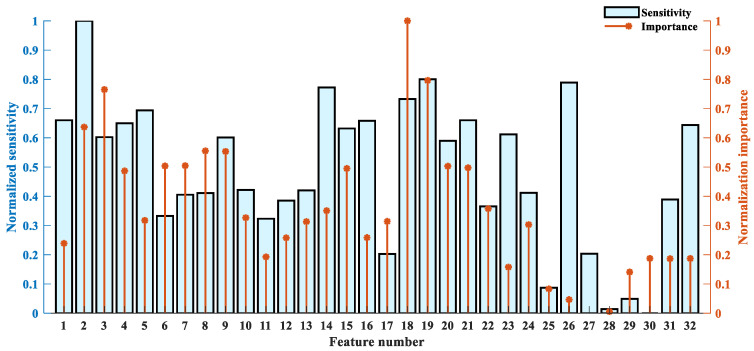
The normalized importance and sensitivity of each feature.

In the RF-mRMR–BCMFDE process, for each feature, the importance ψ and sensitivity Φ were calculated based on Equations (13) and (17), respectively, and the results are shown in Figure 8. In Figure 8, feature indexes 1–16 indicate information about high-frequency component information, and feature indexes 17–32 indicate information about low-frequency component information. It can be found that the high-frequency component contains rich information, which is helpful for classification and compensates for the incomplete feature extraction of the low-frequency components. In addition, the sensitivity and importance of different features have significant differences, and therefore, a weighted feature selection strategy is necessary.

**Figure 9:**
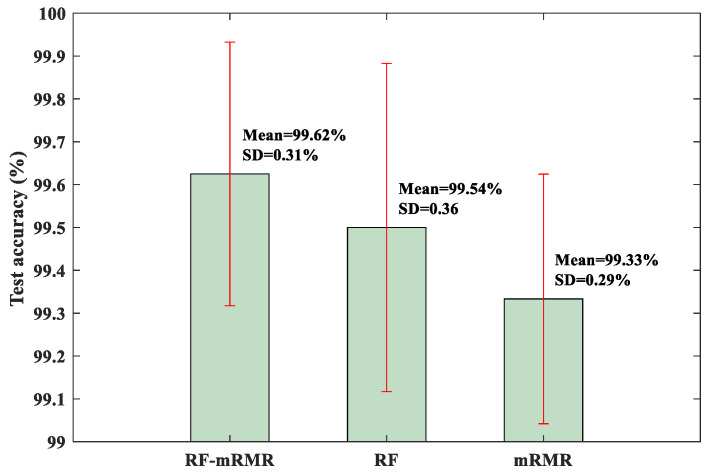
Test accuracy of KNN classifier.

To quantitatively evaluate the RF-mRMR feature selection method, the KNN classifier was trained and tested as before. For comparison, other feature selection methods, including the RF method and the mRMR method, were also used. The test results are shown in Figure 9. From Figure 9, it can be observed that the RF-mRMR method produced the highest mean accuracy and relatively small standard deviation. At the same time, the RF-mRMR method has higher mean accuracy and smaller standard deviation compared with the results of the BCMFDE method (without using the feature selection algorithm) in Table 3. This indicates that the RF-mRMR method can effectively reduce the redundancy of the feature set, further proving the effectiveness of the method.

## 5. Experimental Validation

In this section, two experimental examples are used to verify the bearing damage detection effectiveness and generalization capability of the proposed signal processing framework. The first example is focused on the diagnosis of different fault categories, while the second example emphasizes the fault categories as well as the fault severities.

### 5.1. Example 1: Rolling Bearing Fault Category Identification

#### 5.1.1. Test Setup

**Figure 10:**
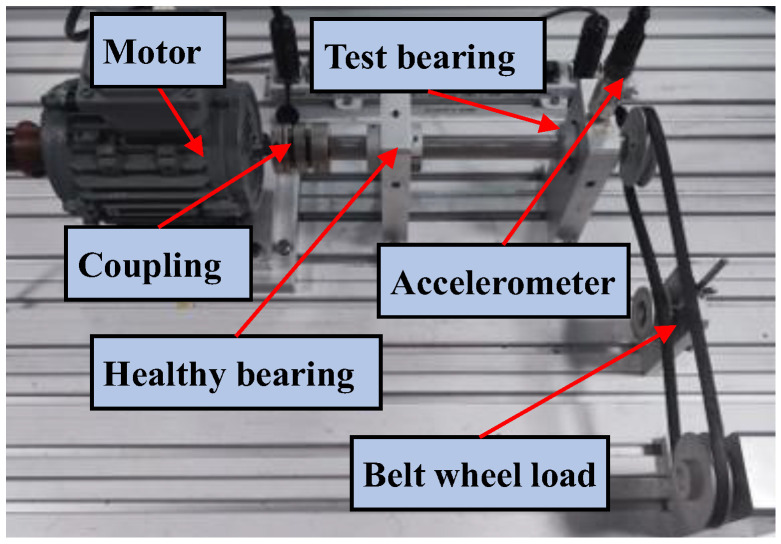
Experimental setup.

The experimental setup is shown in Figure 10, which is composed of a motor, a shaft supported by a test bearing and a healthy bearing, and a belt-wheel loading system. The shaft is driven by the motor at 1800 rpm. The driving side bearing is healthy, and the driven side bearing is the test bearing which can be embedded with different faults. The kinematics parameters of the test bearing are listed in Table 4.

**Figure 11:**
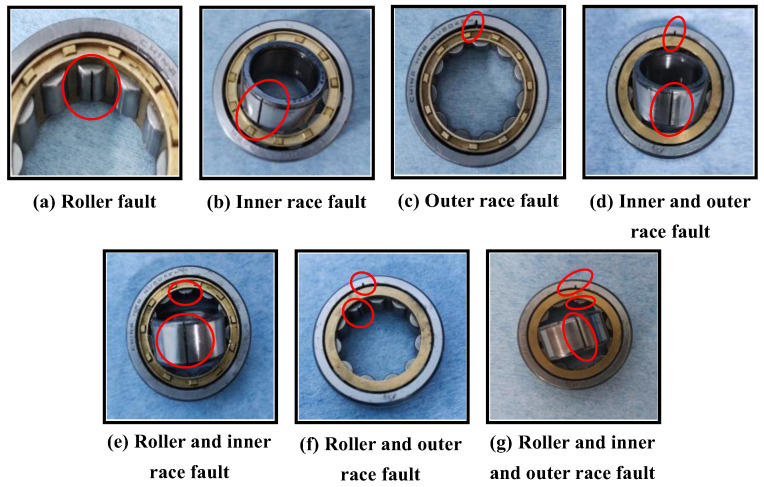
Rolling bearings with artificial pitting defect.

The vibration is picked up by an accelerometer fixed on the test bearing seat and digitized by using an NI9185-based data acquisition system. The sampling frequency is 10.24 kHz, and the sampling duration is 60 s. As shown in Figure 11, three different bearing component faults and three different fault combinations are simulated by artificially introducing damage to the bearing parts with electrical discharge machining (EDM).

**Figure 12:**
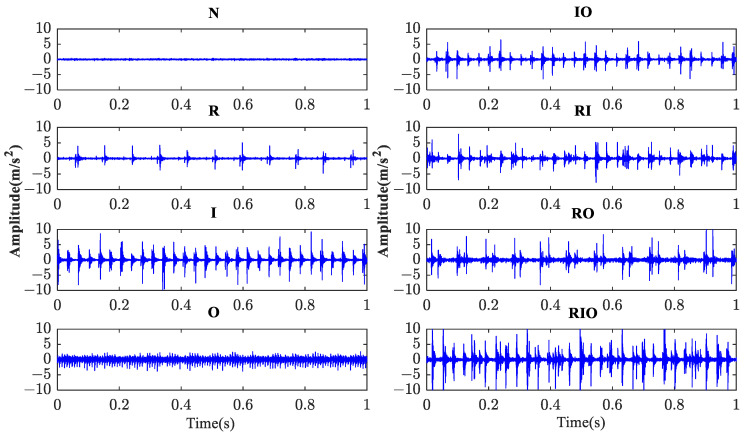
The raw vibration signal of eight categories of conditions of rolling bearings.

Including the healthy baseline, eight categories of bearing conditions were tested, as listed in Table 5. The typical time histories of the vibration signal corresponding to eight bearing categories are shown in Figure 12.

#### 5.1.2. Diagnosis Results and Analysis

The vibration signals corresponding to the eight bearing categories are processed according to the procedure outlined in Section 4. For each bearing vibration response, a sliding window of 2048 points is applied and 300 samples are extracted from the original signal.

**Figure 13:**
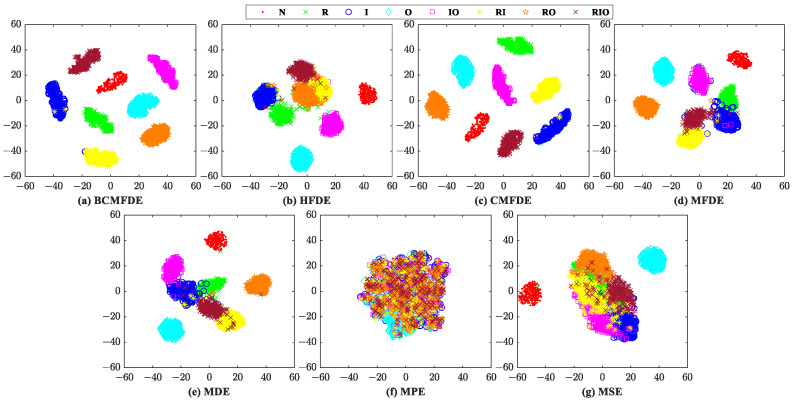
Feature set visualization for different entropy methods.

The evaluation procedure used is similar to the one described in Section 4.2. For comparison purposes, the entropy feature extraction methods based on BCMFDE, HFDE, CMFDE, MFDE, MDE, MPE, and MSE are used. The feature sets constructed by different entropy methods are visualized using the t-SNE algorithm, as shown in Figure 13. According to Figure 13, qualitatively, the feature set constructed by the BCMFDE method has the best separability among all the methods used.

For the quantitative evaluation, the test results of the BCMFDE method and the other entropy feature extraction methods on the KNN classifier are listed in Table 6. It can be seen from Table 6 that the feature extraction method based on BCMFDE obtained the best mean accuracy and the smallest standard deviation.

**Figure 14:**
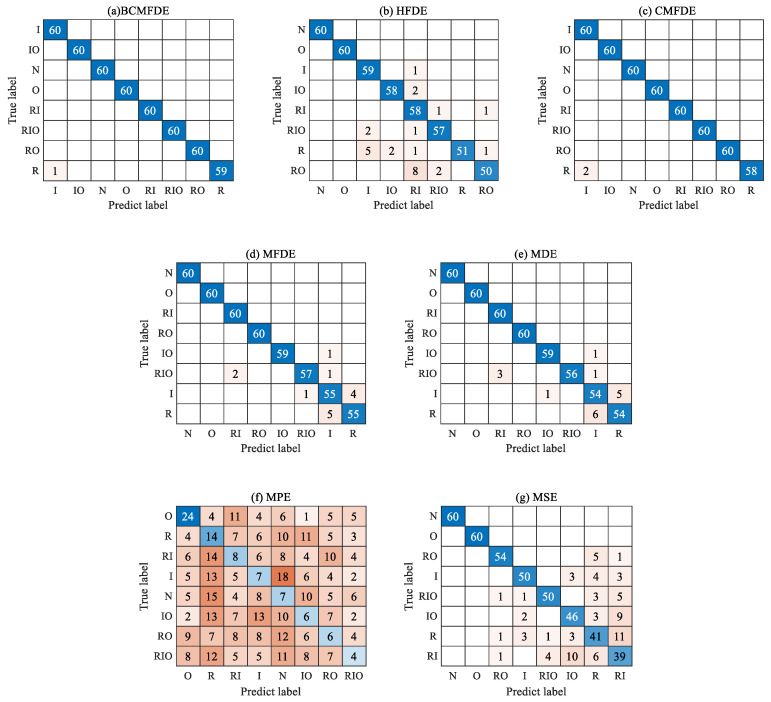
Confusion matrix of different entropy methods.

The confusion matrix of the results of the fifth test was visually analyzed, as shown in Figure 14. As can be seen from Figure 14, the number of misclassified samples is the smallest in the feature set constructed using the BCMFDE method. This indicates that the BCMFDE method has the best feature extraction capability for different fault categories of bearings.

**Figure 15:**
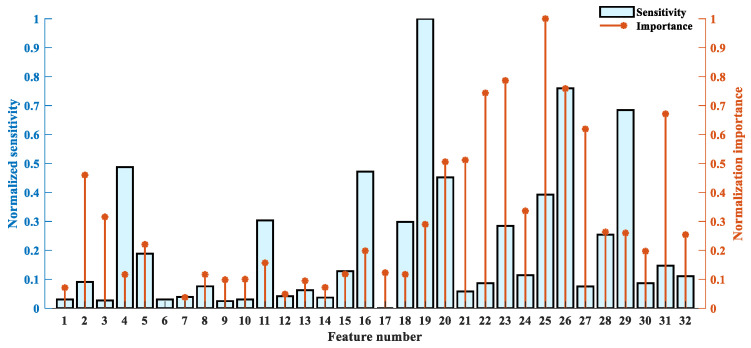
The normalized importance and sensitivity of each feature.

In the RF-mRMR–BCMFDE process, the results of the calculation of importance ψ and sensitivity Φ for each feature are shown in Figure 15. As can be seen in Figure 15, the high-frequency components also contain features of high sensitivity and high importance. In addition, the sensitivity and importance of different features have significant differences and, therefore, suitable feature selection is necessary. In this section, the features with importance ψ and sensitivity Φ in the top τ=16 are selected. The selected features are then used to construct a new feature set.

**Figure 16:**
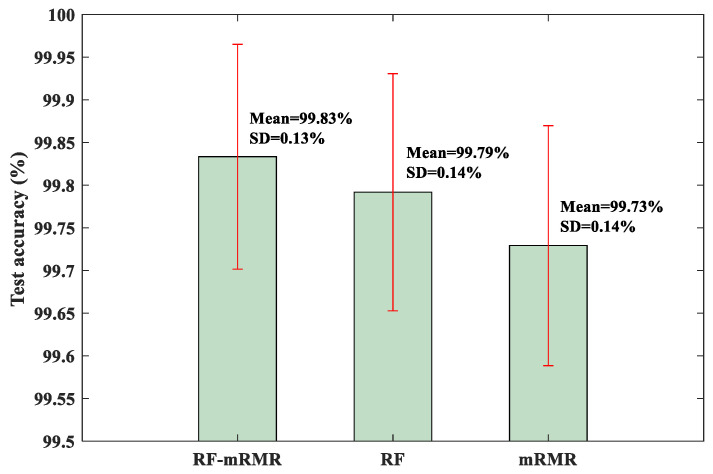
Test accuracy of KNN classifier.

For quantitative evaluation, the test results of the RF-mRMR, RF, and mRMR feature selection methods on the KNN classifier are shown in Figure 16. As can be seen from Figure 16, the RF-mRMR-based feature selection method obtained the best mean accuracy and the smallest standard deviation.

In addition, the RF-mRMR method has the same mean accuracy and standard deviation compared with the results of the BCMFDE method (without using the feature selection algorithm) in Table 6. This demonstrates that the RF-mRMR feature selection method can effectively reduce the redundancy of the feature set without affecting the classification effect.

### 5.2. Example 2: Rolling Bearing Fault Category and Severity Identification

#### 5.2.1. The Test Data

**Figure 17:**
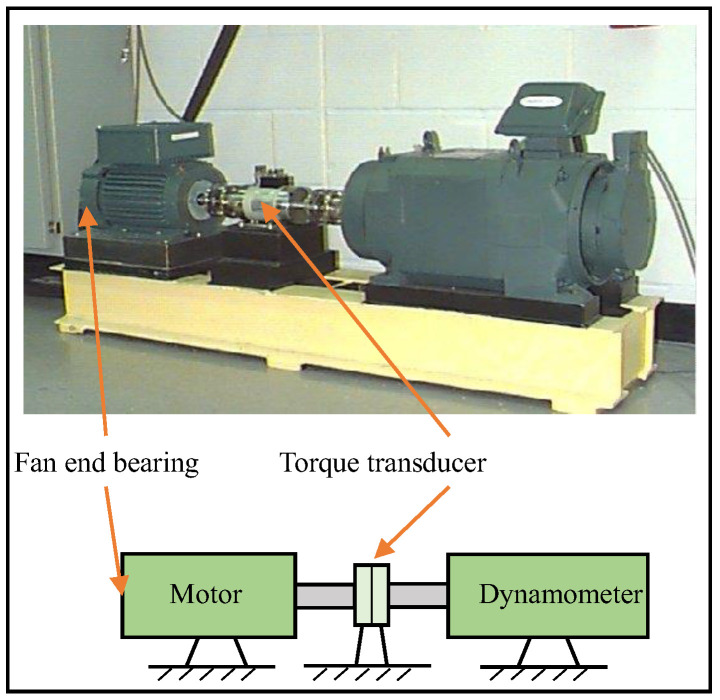
Rolling bearing fault simulation test rig.

In order to verify the effectiveness of the proposed fault diagnosis method in diagnosing different fault categories, as well as the fault severities of rolling bearings, the experimental verification is carried out on the rolling bearing vibration dataset of Case Western Reserve University (CWRU). The rolling bearing fault simulation test rig is shown in Figure 17, which is composed of a motor, a torque transducer, and a dynamometer. The test bearings support the motor shaft. The kinematics parameters of the test bearing are listed in Table 7.

The accelerometer is placed near the drive end of the motor and is used to acquire vibration signals. The vibration signals are collected with a 16-channel DAT recorder. The sampling frequency is 12.00 kHz. Single-point faults are introduced to the test bearing parts using EDM with fault diameters of 0.18 mm, 0.36 mm, and 0.53 mm, respectively. The fault depth is 0.28 mm.

**Figure 18:**
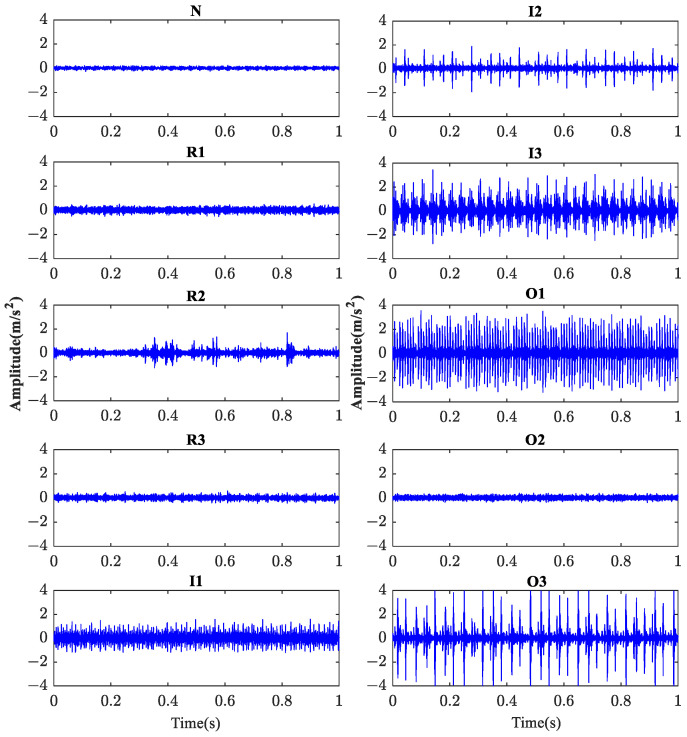
The raw vibration signal of 10 categories of conditions of rolling bearings.

The bearing vibration signal acquired at a motor speed of 1797 rpm is selected as the data sample for analysis. Data samples consist of 10 categories of bearing conditions, including health baseline and different failure categories and severities, as listed in Table 8. The typical time histories of the vibration signals corresponding to the 10 categories of bearings are shown in Figure 18.

#### 5.2.2. Diagnosis Results and Analysis

In this section, the vibration signals corresponding to the 10 categories of bearings obtained in the experiments are processed according to the procedure outlined in Section 4. For each bearing vibration response, a sliding window of 2048 points is applied and 55 samples are extracted from the original signal.

**Figure 19:**
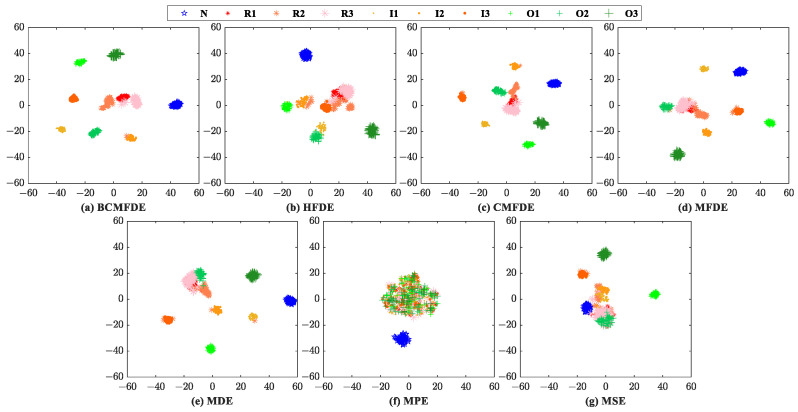
Feature set visualization for different entropy methods.

The evaluation procedure used is similar to Section 4.2. For comparison purposes, the entropy feature extraction methods based on BCMFDE, HFDE, CMFDE, MFDE, MDE, MPE, and MSE are used to extract the entropy features of each sample and construct different feature sets, respectively. The feature sets constructed by different entropy methods are visualized using the t-SNE algorithm, as shown in Figure 19. According to Figure 19, qualitatively, the feature set constructed by the BCMFDE method has the best separability among all the methods used.

For the quantitative evaluation, the test results of the BCMFDE method and the other entropy feature extraction methods on the KNN classifier are listed in Table 9. It can be seen from Table 9 that the feature extraction method based on BCMFDE obtained the best mean accuracy and the smallest standard deviation.

**Figure 20:**
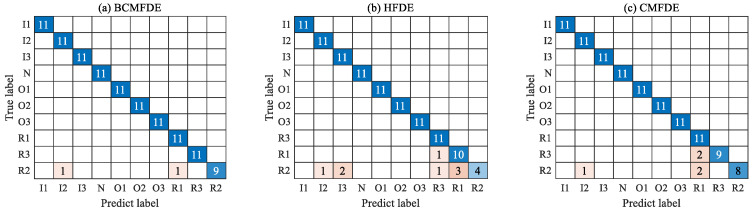

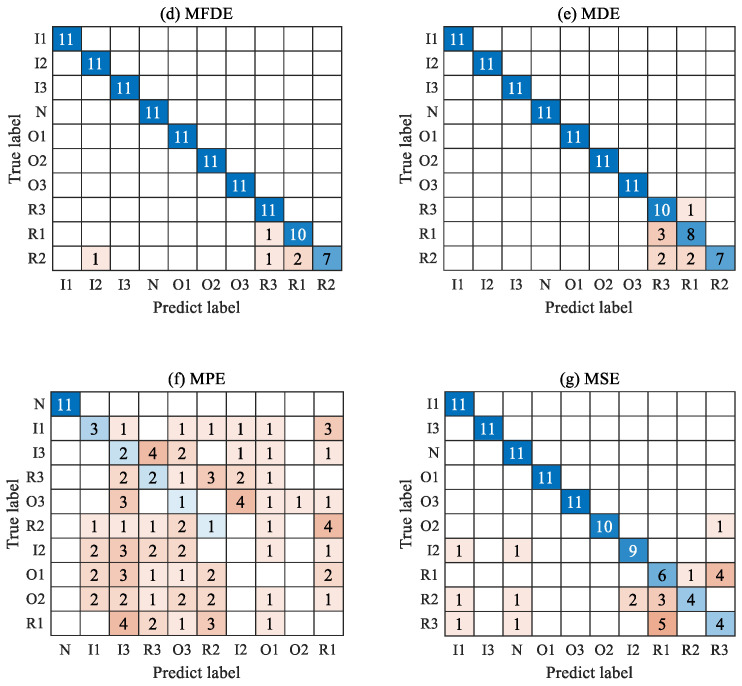
Confusion matrix of different entropy methods.

The confusion matrix of the results of the fifth test was visually analyzed, as shown in Figure 20. As can be seen from Figure 20, the number of misclassified samples is the smallest in the feature set constructed using the BCMFDE method. This indicates that the BCMFDE method has the best feature extraction capability for different fault severities of bearings.

**Figure 21:**
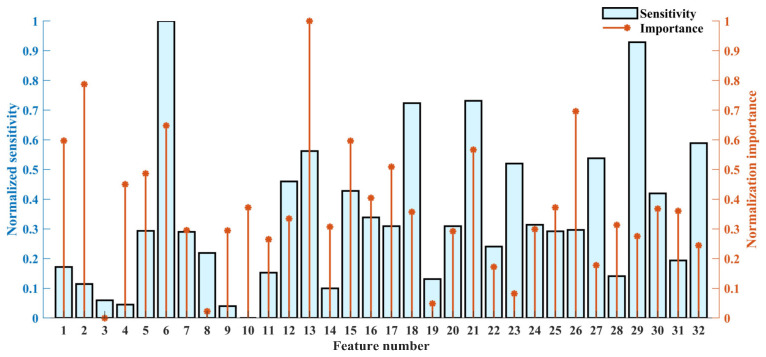
The normalized importance and sensitivity of each feature.

In the RF-mRMR-BCMFDE process, the results of the calculation of importance ψ and sensitivity Φ for each feature are shown in Figure 21. As can be seen in Figure 21, the high-frequency components also contain features of higher sensitivity and importance. This indicates that the high-frequency component information is also important for classification. In addition, the sensitivity and importance of different features have significant differences and, therefore, suitable feature selection is necessary. In this section, the features with importance ψ and sensitivity Φ in the top τ=16 are selected.

**Figure 22:**
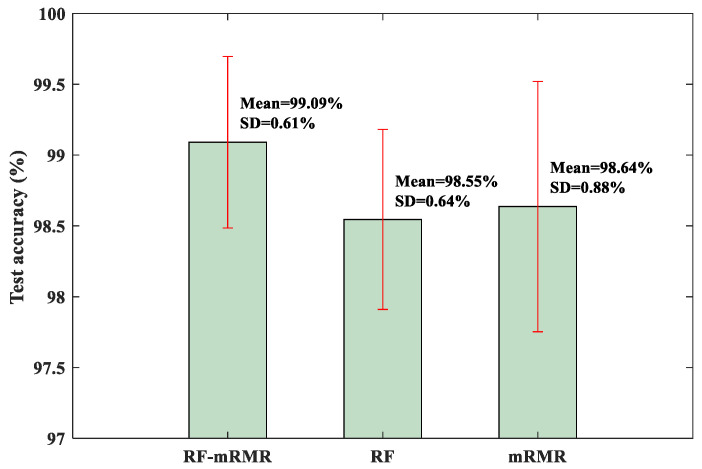
Test accuracy of KNN classifier.

For quantitative evaluation, the test results of the RF-mRMR, RF, and mRMR feature selection methods on the KNN classifier are shown in Figure 22. As can be seen from Figure 22, the RF-mRMR-based feature selection method obtained the best mean accuracy and the smallest standard deviation. At the same time, the RF-mRMR method had a higher mean accuracy and relatively similar standard deviation compared with the results of the BCMFDE method (without using the feature selection algorithm) in Table 9. The validity of the RF-mRMR method was further demonstrated.

## 6. Conclusions

For condition assessment of rolling bearings in rotating machinery, a combination of the BCMFDE-based feature extraction method and RF-mRMR feature selection method is proposed for the construction of rolling bearing fault feature sets. The BCMFDE can extract richer feature information from high-frequency components and low-frequency components for characterizing bearing fault features while The application of RF-mRMR can effectively select features with high importance and sensitivity suitable for classification, thus improving efficiency in the classification and identification of fault categories and reducing the redundancy of the feature sets. The validation results of numerical simulations and two experiments demonstrate that using the proposed framework, i.e., the combination of the fault feature sets construction process and the KNN classifier, is able to automatically identify bearing fault categories, in addition to bearing fault severity. The proposed framework provides a new perspective for intelligent bearing fault diagnosis. With some modifications, it is expected that the framework can be expanded to the intelligent diagnosis of other types of faults such as gear damage or to the intelligent condition monitoring of a complete system.

## Figures and Tables

**Table 1:** Parameters of the simulation bearing.

Parameter	Value
Natural frequency of bearing	4000 Hz
Pitch diameter	34 mm
Roller diameter	7.5 mm
Number of rollers	11
Contact angle	0°

**Table 2:** Parameters of the entropy-based methods.

Entropy	Dimension *m*	Classes *c*	Delay *d*	Tolerance *r*	Scale τ	Layer k
BCMFDE	2	5	1	-	16	-
CMFDE						
HFDE					-	4
MFDE					16	-
MDE						
MPE		-				
MSE			-	0.25 std		

**Table 3:** Testing accuracy and time obtained using different methods.

Different Methods	Number of Tests										Mean	SD	Time (s)
	1	2	3	4	5	6	7	8	9	10			
BCMFDE	98.33%	99.17%	99.17%	98.75%	98.75%	98.75%	99.17%	99.17%	98.75%	99.58%	98.96%	0.35%	45.92
HFDE	83.33%	86.25%	90.00%	87.08%	86.25%	86.67%	87.92%	84.58%	86.67%	85.42%	86.42%	1.81%	131.01
CMFDE	97.08%	97.92%	97.92%	96.25%	97.50%	99.17%	98.33%	97.92%	97.50%	98.75%	97.83%	0.83%	23.92
MFDE	92.5%	94.58%	95.00%	92.50%	94.17%	94.58%	94.17%	94.17%	93.33%	94.58%	93.96%	0.88%	4.60
MDE	90.83%	93.75%	94.58%	92.92%	90.83%	92.50%	92.08%	93.75%	92.08%	95.83%	92.92%	1.60%	1.31
MPE	28.75%	23.75%	30.42%	29.58%	30.00%	19.58%	25.83%	30.00%	25.83%	30.83%	27.46%	3.66%	4.75
MSE	89.17%	90.00%	92.08%	92.50%	90.42%	92.50%	92.08%	94.58%	88.75%	92.50%	91.46%	1.81%	239.59

**Table 4:** Parameters of the test bearing.

Parameter	Value
Bearing type	NU204 ECP
Pitch diameter	34 mm
Roller diameter	7.5 mm
Number of rollers	11
Contact angle	0°

**Table 5:** Tested bearings.

Bearing State	Abbreviation
Normal	N
Roller fault	R
Inner race fault	I
Outer race fault	O
Inner and outer race fault	IO
Roller and inner race fault	RI
Roller and outer race fault	RO
Roller and inner and outer race fault	RIO

**Table 6:** Testing accuracy and time obtained using different methods.

Different Methods	Number of Tests										Mean	SD	Time (s)
	1	2	3	4	5	6	7	8	9	10			
BCMFDE	99.79%	99.79%	100%	99.79%	99.79%	100%	100%	99.79%	99.58%	99.79%	99.83%	0.13%	91.93
HFDE	93.75%	94.58%	95.21%	96.46%	94.38%	94.79%	97.08%	95.63%	96.04%	95.83%	95.37%	1.02%	292.82
CMFDE	99.79%	99.79%	100%	99.79%	99.58%	99.79%	100%	99.58%	99.58%	99.79%	99.77%	0.15%	76.46
MFDE	97.71%	97.92%	97.71%	96.88%	97.08%	97.29%	97.92%	98.13%	97.92%	96.46%	97.50%	0.55%	8.81
MDE	96.46%	96.25%	97.50%	96.25%	96.46%	96.88%	97.08%	97.50%	96.88%	95.63%	96.69%	0.59%	2.61
MPE	15.83%	16.67%	17.29%	17.29%	15.83%	16.25%	17.71%	14.79%	16.67%	19.58%	16.79%	1.30%	9.72
MSE	84.38%	84.17%	83.33%	86.04%	83.33%	84.17%	85.83%	82.29%	82.92%	83.75%	84.02%	1.19%	308.12

**Table 7:** Parameters of the test bearing.

Parameter	Value
Bearing type	6205–2RS JEM SKF
Pitch diameter	39.04 (mm)
Roller diameter	7.94 (mm)
Number of the roller	9
Contact angle	0°

**Table 8:** The detailed descriptions for 10 different working conditions.

Bearing State	Defect Size (mm)	Abbreviation
Normal	0	N
Roller fault	0.18	R1
Roller fault	0.36	R2
Roller fault	0.53	R3
Inner race fault	0.18	I1
Inner race fault	0.36	I2
Inner race fault	0.53	I3
Outer race fault	0.18	O1
Outer race fault	0.36	O2
Outer race fault	0.53	O3

**Table 9:** Testing accuracy and time obtained using different methods.

Different Methods	Number of Tests										Mean	SD	Time (s)
	1	2	3	4	5	6	7	8	9	10			
BCMFDE	98.18%	98.18%	98.18%	98.18%	98.18%	98.18%	99.09%	99.09%	98.18%	97.27%	98.27%	0.52%	21.30
HFDE	94.55%	92.73%	93.64%	95.45%	92.73%	96.36%	90.90%	95.45%	93.64%	95.45%	94.09%	1.67%	54.83
CMFDE	96.36%	95.45%	98.18%	98.18%	95.45%	97.27%	97.27%	99.09%	96.36%	97.27%	97.09%	1.20%	10.72
MFDE	94.55%	95.45%	94.55%	94.55%	95.45%	96.36%	98.18%	95.45%	96.36%	93.64%	95.45%	1.29%	1.98
MDE	93.64%	91.82%	90.91%	91.82%	92.73%	96.36%	98.18%	91.82%	88.18%	90.91%	92.64%	2.86%	0.63
MPE	19.09%	20.91%	19.09%	19.09%	18.18%	24.55%	20.91%	23.64%	19.09%	19.09%	20.36%	2.15%	2.24
MSE	79.09%	80.91%	80.00%	82.73%	80.00%	81.82%	84.55%	85.45%	82.73%	80.00%	81.73%	2.12%	71.63

## Data Availability

The data used to support the findings of this study are available from the corresponding author upon request.

## References

[1] Jardine A.K.S., Lin D., Banjevic D. (2006). A review on machinery diagnostics and prognostics implementing condition-based maintenance. Mech. Syst. Signal. Process..

[2] Zhang X., Miao Q., Zhang H., Wang L. (2018). A parameter-adaptive VMD method based on grasshopper optimization algorithm to analyze vibration signals from rotating machinery. Mech. Syst. Signal. Process..

[3] Cerrada M., Sanchez R.V., Li C., Pacheco F., Cabrera D., Oliveira J.V., Vsquez R.E. (2018). A review on data-driven fault severity assessment in rolling bearings. Mech. Syst. Signal. Process..

[4] Wang D., Tsui K.L. (2017). Statistical Modeling of Bearing Degradation Signals. IEEE Trans. Reliab..

[5] Tandon N., Choudhury A. (1999). A review of vibration and acoustic measurement methods for the detection of defects in rolling element bearings. Tribol. Int..

[6] Wang B., Tao F., Fang X., Liu C., Liu Y., Freiheit T. (2021). Smart Manufacturing and Intelligent Manufacturing: A Comparative Review. Engineering.

[7] Saufi S., Ahmad Z., Leong M., Lim M. (2019). An intelligent bearing fault diagnosis system: A review. MATEC Web Conf..

[8] Zhang T., Chen J., Li F., Zhang K., Lv H., He S., Xu E. (2022). Intelligent fault diagnosis of machines with small & imbalanced data: A state-of-the-art review and possible extensions. ISA Trans..

[9] Rai A., Upadhyay S. (2016). A review on signal processing techniques utilized in the fault diagnosis of rolling element bearings. Tribol. Int..

[10] Shannon C. (1948). Mathematical Theory of Communication. Bell Syst. Tech. J..

[11] Yan R., Gao R. (2007). Approximate Entropy as a diagnostic tool for machine health monitoring. Mech. Syst. Signal. Process..

[12] Han M., Pan J. (2015). A fault diagnosis method combined with LMD, sample entropy and energy ratio for roller bearings. Measurement.

[13] Zheng J., Cheng J., Yang Y. (2013). A rolling bearing fault diagnosis approach based on LCD and fuzzy entropy. Mech. Mach. Theory.

[14] Zhang X., Liang Y., Zhou J. (2015). A novel bearing fault diagnosis model integrated permutation entropy, ensemble empirical mode decomposition and optimized SVM. Measurement.

[15] Rostaghi M., Ashory M., Azami H. (2019). Application of dispersion entropy to status characterization of rotary machines. J. Sound Vib..

[16] Li Y., Geng B., Tang B. (2023). Simplified coded dispersion entropy: A nonlinear metric for signal analysis. Nonlinear Dyn..

[17] Li Y., Gao X., Wang L. (2019). Reverse Dispersion Entropy: A New Complexity Measure for Sensor Signal. Sensors.

[18] Costa M., Goldberger A., Peng C. (2002). Multiscale Entropy Analysis of Complex Physiologic Time Series. Phys. Rev. Lett..

[19] Zhang L., Xiong G., Liu H., Zou H., Guo W. (2010). Bearing fault diagnosis using multi-scale entropy and adaptive neuro-fuzzy inference. Expert Syst. Appl..

[20] Wu S., Wu P., Wu C., Ding J., Wang C. (2012). Bearing Fault Diagnosis Based on Multiscale Permutation Entropy and Support Vector Machine. Entropy.

[21] Zhang Y., Tong S., Cong F., Xu J. (2018). Research of Feature Extraction Method Based on Sparse Reconstruction and Multiscale Dispersion Entropy. Appl. Sci..

[22] Wu S., Wu C., Lin S., Wang C., Lee K. (2013). Time Series Analysis Using Composite Multiscale Entropy. Entropy.

[23] Jiang Y., Peng C., Xu Y. (2011). Hierarchical entropy analysis for biological signals. J. Comput. Appl. Math..

[24] Li Y., Wang X., Zheng J., Feng K., Ji J. (2023). Bi-filter multiscale-diversity-entropy-based weak feature extraction for a rotor-bearing system. Meas. Sci. Technol..

[25] Rostaghi M., Khatibi M.M., Ashory M.R., Azami H. (2022). Fuzzy Dispersion Entropy: A Nonlinear Measure for Signal Analysis. IEEE Trans. Fuzzy Syst..

[26] Li Y., Wu J., Zhang S., Tang B., Lou Y. (2023). Variable-Step Multiscale Fuzzy Dispersion Entropy: A Novel Metric for Signal Analysis. Entropy.

[27] Yan X., Jia M. (2019). Intelligent fault diagnosis of rotating machinery using improved multiscale dispersion entropy and mRMR feature selection. Knowl. Based Syst..

[28] Liaw A., Wiener M. (2002). Classification and Regression by random Forest. R News.

[29] Liu W., Zheng Y., Zhou X., Chen Q. (2023). Axis Orbit Recognition of the Hydropower Unit Based on Feature Combination and Feature Selection. Sensors.

[30] Wang X., Si S., Li Y. (2022). Hierarchical diversity entropy for the early fault diagnosis of rolling bearing. Nonlinear Dyn..

[31] Zhang X., Song Z., Li D., Zhang W., Zhao Z., Chen Y. (2018). Fault diagnosis for reducer via improved LMD and SVM-RFE-MRMR. Shock. Vib..

[32] Hu Q., Si X., Qin A., Lv Y., Zhang Q. (2020). Machinery Fault Diagnosis Scheme Using Redefined Dimensionless Indicators and mRMR Feature Selection. IEEE Access.

[33] Ding C., Peng H. (2005). Minimum redundancy feature selection from microarray gene expression data. J. Bioinf. Comput. Biol..

[34] Lu Q., Shen X., Wang X., Li M., Li J., Zhang M. (2021). Fault Diagnosis of Rolling Bearing Based on Improved VMD and KNN. Math. Probl. Eng..

[35] Randall R.B., Antoni J. (2011). Rolling element bearing diagnostics—A tutorial. Mech. Syst. Signal. Process..

[36] Gan X., Lu H., Yang G. (2019). Fault Diagnosis Method for Rolling Bearings Based on Composite Multiscale Fluctuation Dispersion Entropy. Entropy.

[37] Van der Maaten L., Hinton G. (2008). Visualizing data using t-SNE. J. Mach. Learn. Res..

